# Supplementary material for: StressME: Unified computing framework of Escherichia coli metabolism, gene expression, and stress responses
Source: PLoS Comput Biol. 2024 Feb 12;20(2):e1011865. doi: 10.1371/journal.pcbi.1011865 (PMC10890762; doi:10.1371/journal.pcbi.1011865)
Supplement: S1 Appendix — (DOCX) [file pcbi.1011865.s001.docx]

**S1 Appendix: Biological expansion of StressME**

**Table A. Difference in model components between StressME and EcoliME**

| **Model components** | **StressME** | **EcoliME** |
| --- | --- | --- |
| **genes** | 1,689 | 1,678 |
| **mRNAs** | 1,689 | 1,678 |
| **tRNAs** | 63 | 63 |
| **proteins** | 1,578 | 1,568 |
| **protein folding states** | 9,561 | 0 |
| **metabolites** | 1,673 | 1,671 |
| **complexes** | 1,692 | 1,526 |
| **reactions** | 36,735 | 12,655 |

**Table B. Reactions added to StressME after every reconstruction step**

| **Steps** | **Added reactions*** |
| --- | --- |
| 1: EcoliME (iJL1678b) => FoldME (℃) | 21,957 |
| 2: FoldME (℃) => StressME (℃) | 177 |
| 3: StressME (℃) => StressME (℃, pH) | 217 |
| 4: StressME (℃, pH) => StressME (℃, pH, ROS) | 1,729 |

*Detailed information about the reactions with chemical equations can be found in S2 Appendix; 24,080 more reactions have been added to the original EcoliME (iJL1678b) to generate StressME.
