# Supplementary material for: StressME: Unified computing framework of Escherichia coli metabolism, gene expression, and stress responses
Source: PLoS Comput Biol. 2024 Feb 12;20(2):e1011865. doi: 10.1371/journal.pcbi.1011865 (PMC10890762; doi:10.1371/journal.pcbi.1011865)
Supplement: S3 Appendix — (DOCX) [file pcbi.1011865.s003.docx]

**S3 Appendix: Temperature-dependent thermostability, aggregation propensity, and folding rate constants for ten folding proteins added to StressME.**

**Table A. Temperature-dependent thermostability (Oobatake Keq [1]) for 10 folding proteins added to StressME**

|  | **b0605** | **b0606** | **b2962** | **b4209** | **b3662** | **b0812** | **b3961** | **b4062** | **b4063** | **b0439** |
| --- | --- | --- | --- | --- | --- | --- | --- | --- | --- | --- |
| **26℃** | 5.0E-12 | 4.2E+03 | 7.1E-07 | 8.6E+00 | 1.1E-29 | 8.4E+02 | 2.1E-04 | 2.1E-02 | 1.4E+07 | 9.5E+31 |
| **27℃** | 8.0E-12 | 6.2E+03 | 8.4E-07 | 1.1E+01 | 4.4E-29 | 8.9E+02 | 3.0E-04 | 2.1E-02 | 1.2E+07 | 8.1E+31 |
| **28℃** | 1.3E-11 | 9.6E+03 | 1.0E-06 | 1.4E+01 | 1.8E-28 | 9.7E+02 | 4.4E-04 | 2.2E-02 | 1.1E+07 | 7.5E+31 |
| **29℃** | 2.2E-11 | 1.6E+04 | 1.2E-06 | 1.8E+01 | 7.4E-28 | 1.1E+03 | 6.7E-04 | 2.3E-02 | 9.7E+06 | 7.6E+31 |
| **30℃** | 3.8E-11 | 2.7E+04 | 1.5E-06 | 2.4E+01 | 3.2E-27 | 1.2E+03 | 1.1E-03 | 2.4E-02 | 8.8E+06 | 8.4E+31 |
| **31℃** | 6.6E-11 | 4.8E+04 | 1.8E-06 | 3.2E+01 | 1.5E-26 | 1.4E+03 | 1.7E-03 | 2.6E-02 | 8.1E+06 | 1.0E+32 |
| **32℃** | 1.2E-10 | 9.1E+04 | 2.2E-06 | 4.5E+01 | 6.9E-26 | 1.6E+03 | 2.9E-03 | 2.8E-02 | 7.5E+06 | 1.3E+32 |
| **33℃** | 2.1E-10 | 1.8E+05 | 2.7E-06 | 6.3E+01 | 3.4E-25 | 1.9E+03 | 5.0E-03 | 3.0E-02 | 7.2E+06 | 1.8E+32 |
| **34℃** | 3.7E-10 | 3.7E+05 | 3.4E-06 | 9.1E+01 | 1.7E-24 | 2.3E+03 | 8.9E-03 | 3.4E-02 | 6.9E+06 | 2.8E+32 |
| **35℃** | 6.9E-10 | 8.1E+05 | 4.3E-06 | 1.3E+02 | 9.1E-24 | 2.8E+03 | 1.6E-02 | 3.8E-02 | 6.8E+06 | 4.5E+32 |
| **36℃** | 1.3E-09 | 1.8E+06 | 5.4E-06 | 2.0E+02 | 5.0E-23 | 3.4E+03 | 3.1E-02 | 4.3E-02 | 6.7E+06 | 8.0E+32 |
| **37℃** | 2.4E-09 | 4.4E+06 | 6.9E-06 | 3.1E+02 | 2.8E-22 | 4.3E+03 | 5.9E-02 | 4.9E-02 | 6.8E+06 | 1.5E+33 |
| **38℃** | 4.7E-09 | 1.1E+07 | 8.9E-06 | 4.9E+02 | 1.7E-21 | 5.5E+03 | 1.2E-01 | 5.7E-02 | 7.0E+06 | 3.1E+33 |
| **39℃** | 9.1E-09 | 2.9E+07 | 1.2E-05 | 7.8E+02 | 1.0E-20 | 7.2E+03 | 2.4E-01 | 6.6E-02 | 7.2E+06 | 6.8E+33 |
| **40℃** | 1.8E-08 | 7.9E+07 | 1.5E-05 | 1.3E+03 | 6.3E-20 | 9.5E+03 | 5.1E-01 | 7.8E-02 | 7.6E+06 | 1.6E+34 |
| **41℃** | 3.6E-08 | 2.2E+08 | 2.0E-05 | 2.1E+03 | 4.1E-19 | 1.3E+04 | 1.1E+00 | 9.4E-02 | 8.2E+06 | 4.1E+34 |
| **42℃** | 7.4E-08 | 6.7E+08 | 2.6E-05 | 3.5E+03 | 2.8E-18 | 1.7E+04 | 2.4E+00 | 1.1E-01 | 8.9E+06 | 1.1E+35 |
| **43℃** | 1.5E-07 | 2.1E+09 | 3.5E-05 | 6.1E+03 | 1.9E-17 | 2.4E+04 | 5.6E+00 | 1.4E-01 | 9.8E+06 | 3.3E+35 |
| **44℃** | 3.2E-07 | 6.7E+09 | 4.7E-05 | 1.1E+04 | 1.4E-16 | 3.3E+04 | 1.3E+01 | 1.7E-01 | 1.1E+07 | 1.0E+36 |
| **45℃** | 6.7E-07 | 2.3E+10 | 6.3E-05 | 1.9E+04 | 1.0E-15 | 4.7E+04 | 3.1E+01 | 2.1E-01 | 1.2E+07 | 3.5E+36 |
| **46℃** | 1.4E-06 | 7.9E+10 | 8.6E-05 | 3.4E+04 | 8.0E-15 | 6.7E+04 | 7.7E+01 | 2.6E-01 | 1.4E+07 | 1.2E+37 |

**Table B. Aggregation propensity for 10 folding proteins added to StressME**

|  | **propensity** | **confidence** |
| --- | --- | --- |
| **b0605** | 5 | 5.29 |
| **b0606** | 12 | 5.19 |
| **b2962** | 2 | 7.31 |
| **b4209** | 2 | 5.54 |
| **b3662** | 14 | 5.60 |
| **b0812** | 6 | 5.65 |
| **b3961** | 9 | 5.18 |
| **b4062** | 3 | 5.14 |
| **b4063** | 3 | 6.21 |
| **b0439** | 11 | 5.12 |

**Table C. Temperature-dependent folding rate constants for ten folding proteins added to StressME**

|  | **b0605** | **b0606** | **b2962** | **b4209** | **b3662** | **b0812** | **b3961** | **b4062** | **b4063** | **b0439** |
| --- | --- | --- | --- | --- | --- | --- | --- | --- | --- | --- |
| **26℃** | 3.2E-02 | 1.4E+00 | 1.6E-02 | 1.8E+04 | 8.1E-03 | 2.5E+03 | 2.5E+04 | 1.9E+03 | 6.1E+04 | 1.9E+01 |
| **27℃** | 4.1E-02 | 1.7E+00 | 2.1E-02 | 2.3E+04 | 1.0E-02 | 3.2E+03 | 3.2E+04 | 2.4E+03 | 7.7E+04 | 2.4E+01 |
| **28℃** | 5.2E-02 | 2.2E+00 | 2.7E-02 | 2.9E+04 | 1.3E-02 | 4.0E+03 | 4.1E+04 | 3.1E+03 | 9.9E+04 | 3.1E+01 |
| **29℃** | 6.6E-02 | 2.8E+00 | 3.4E-02 | 3.7E+04 | 1.7E-02 | 5.2E+03 | 5.2E+04 | 3.9E+03 | 1.3E+05 | 3.9E+01 |
| **30℃** | 8.4E-02 | 3.6E+00 | 4.3E-02 | 4.7E+04 | 2.1E-02 | 6.6E+03 | 6.6E+04 | 5.0E+03 | 1.6E+05 | 5.0E+01 |
| **31℃** | 1.1E-01 | 4.6E+00 | 5.5E-02 | 6.0E+04 | 2.7E-02 | 8.3E+03 | 8.3E+04 | 6.4E+03 | 2.0E+05 | 6.3E+01 |
| **32℃** | 1.3E-01 | 5.8E+00 | 7.0E-02 | 7.6E+04 | 3.4E-02 | 1.1E+04 | 1.1E+05 | 8.1E+03 | 2.6E+05 | 8.0E+01 |
| **33℃** | 1.7E-01 | 7.3E+00 | 8.8E-02 | 9.7E+04 | 4.3E-02 | 1.3E+04 | 1.3E+05 | 1.0E+04 | 3.3E+05 | 1.0E+02 |
| **34℃** | 2.2E-01 | 9.3E+00 | 1.1E-01 | 1.2E+05 | 5.5E-02 | 1.7E+04 | 1.7E+05 | 1.3E+04 | 4.1E+05 | 1.3E+02 |
| **35℃** | 2.7E-01 | 1.2E+01 | 1.4E-01 | 1.5E+05 | 6.9E-02 | 2.1E+04 | 2.1E+05 | 1.6E+04 | 5.2E+05 | 1.6E+02 |
| **36℃** | 3.4E-01 | 1.5E+01 | 1.8E-01 | 1.9E+05 | 8.7E-02 | 2.7E+04 | 2.7E+05 | 2.1E+04 | 6.5E+05 | 2.0E+02 |
| **37℃** | 4.3E-01 | 1.9E+01 | 2.2E-01 | 2.4E+05 | 1.1E-01 | 3.4E+04 | 3.4E+05 | 2.6E+04 | 8.2E+05 | 2.5E+02 |
| **38℃** | 5.4E-01 | 2.3E+01 | 2.8E-01 | 3.1E+05 | 1.4E-01 | 4.2E+04 | 4.2E+05 | 3.2E+04 | 1.0E+06 | 3.2E+02 |
| **39℃** | 6.8E-01 | 2.9E+01 | 3.5E-01 | 3.8E+05 | 1.7E-01 | 5.3E+04 | 5.3E+05 | 4.1E+04 | 1.3E+06 | 4.0E+02 |
| **40℃** | 8.5E-01 | 3.7E+01 | 4.4E-01 | 4.8E+05 | 2.2E-01 | 6.6E+04 | 6.7E+05 | 5.1E+04 | 1.6E+06 | 5.0E+02 |
| **41℃** | 1.1E+00 | 4.6E+01 | 5.5E-01 | 6.0E+05 | 2.7E-01 | 8.3E+04 | 8.3E+05 | 6.4E+04 | 2.0E+06 | 6.3E+02 |
| **42℃** | 1.3E+00 | 5.7E+01 | 6.9E-01 | 7.5E+05 | 3.4E-01 | 1.0E+05 | 1.0E+06 | 8.0E+04 | 2.5E+06 | 7.9E+02 |
| **43℃** | 1.7E+00 | 7.1E+01 | 8.6E-01 | 9.4E+05 | 4.2E-01 | 1.3E+05 | 1.3E+06 | 9.9E+04 | 3.2E+06 | 9.8E+02 |
| **44℃** | 2.1E+00 | 8.9E+01 | 1.1E+00 | 1.2E+06 | 5.2E-01 | 1.6E+05 | 1.6E+06 | 1.2E+05 | 3.9E+06 | 1.2E+03 |
| **45℃** | 2.6E+00 | 1.1E+02 | 1.3E+00 | 1.5E+06 | 6.5E-01 | 2.0E+05 | 2.0E+06 | 1.5E+05 | 4.9E+06 | 1.5E+03 |
| **46℃** | 3.2E+00 | 1.4E+02 | 1.7E+00 | 1.8E+06 | 8.1E-01 | 2.5E+05 | 2.5E+06 | 1.9E+05 | 6.1E+06 | 1.9E+03 |

1. Oobatake M, Ooi T. Hydration and heat stability effects on protein unfolding. Prog Biophys Mol Biol. 1993;59(3):237-84. doi: 10.1016/0079-6107(93)90002-2. PubMed PMID: 8441810.
