# Supplementary material for: StressME: Unified computing framework of Escherichia coli metabolism, gene expression, and stress responses
Source: PLoS Comput Biol. 2024 Feb 12;20(2):e1011865. doi: 10.1371/journal.pcbi.1011865 (PMC10890762; doi:10.1371/journal.pcbi.1011865)
Supplement: S4 Appendix — (DOCX) [file pcbi.1011865.s004.docx]

**S4 Appendix: Typical computation time for running StressME**

**Table A. Typical computation time for running relevant use cases of StressME in Graham clusters**

| **Stress conditions**  **(multiple stress)** | **Task***  **(Objective)** | **Function**  **(MINOS)** | **Cold-start**  **(minutes)** | **Warm-start**  **(minutes)** |
| --- | --- | --- | --- | --- |
| 37℃, pH 7.0, ROS 1x | Max growth | bisectmu( ) | 42.1 | 8.9 |
| 42℃, pH 5.0, ROS 10x | Max growth | bisectmu( ) | 140.0 | 36.7 |
| 37℃, pH 7.0, ROS 1x | Max acetate**(fixed growth) | Solvelp( ) | 21.4 | 0.8 |
| 42℃, pH 5.0, ROS 10x | Max acetate**(fixed growth) | Solvelp( ) | 28.4 | 3.9 |

***** Reaction to be maximized can be growth rate, metabolic or transcription, translation, complex formation rates, etc.

** When multiple conditions are simulated, it will be much more efficient to run the simulations in parallel on a cluster.

**Table B. Typical computation time for running relevant use cases of StressME in local computer by docker**

| **Stress conditions**  **(multiple stress)** | **Task***  **(Objective)** | **Function**  **(MINOS)** | **Cold-start**  **(minutes)** | **Warm-start**  **(minutes)** |
| --- | --- | --- | --- | --- |
| 37℃, pH 7.0, ROS 1x | Max growth | bisectmu( ) | 52.7 | 14.0 |
| 42℃, pH 5.0, ROS 10x | Max growth | bisectmu( ) | 175.7 | 46.1 |
| 37℃, pH 7.0, ROS 1x | Max acetate**(fixed growth) | Solvelp( ) | 37.9 | 2.9 |
| 42℃, pH 5.0, ROS 10x | Max acetate**(fixed growth) | Solvelp( ) | 44.8 | 5.6 |

***** Reaction to be maximized can be growth rate, metabolic or transcription, translation, complex formation rates, etc.
