# Supplementary material for: StressME: Unified computing framework of Escherichia coli metabolism, gene expression, and stress responses
Source: PLoS Comput Biol. 2024 Feb 12;20(2):e1011865. doi: 10.1371/journal.pcbi.1011865 (PMC10890762; doi:10.1371/journal.pcbi.1011865)
Supplement: S5 Appendix — (DOCX) [file pcbi.1011865.s005.docx]

**S5 Appendix: Material balance check for metabolites and proteome


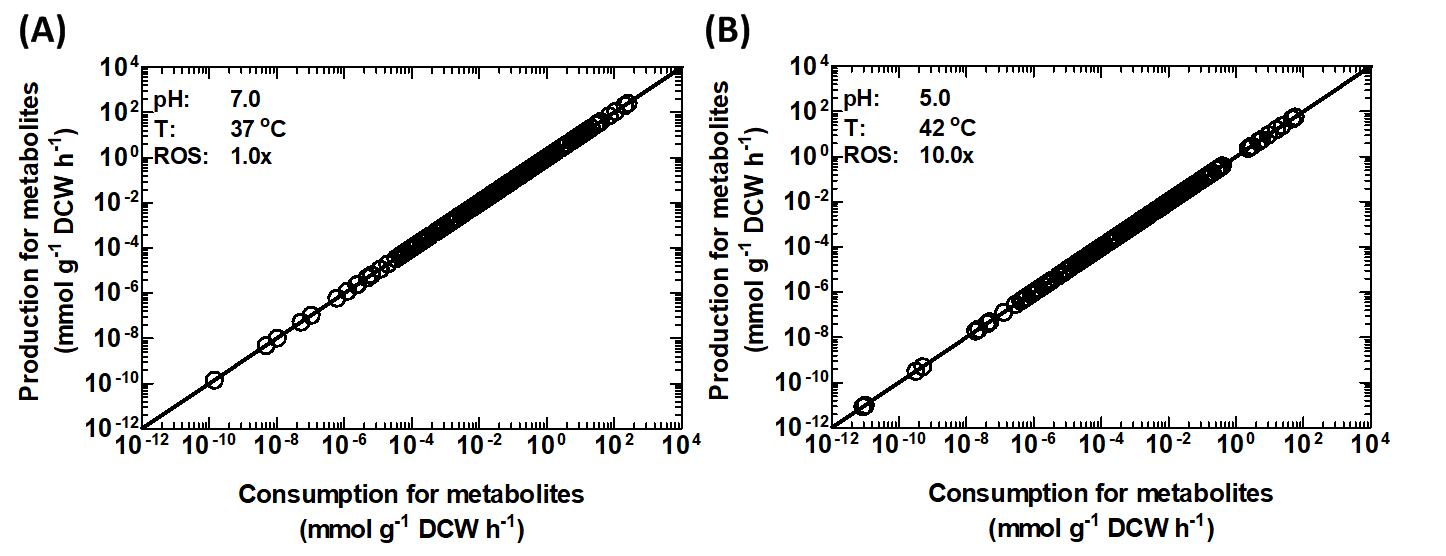
**

Fig A. Mass balance check for all 1673 metabolites in StressME by consumption and production of each metabolite under representative conditions (wild type strain, 37 ℃-pH7.0-ROS1.0x and 42 ℃-pH5.0-ROS10.0x)

**
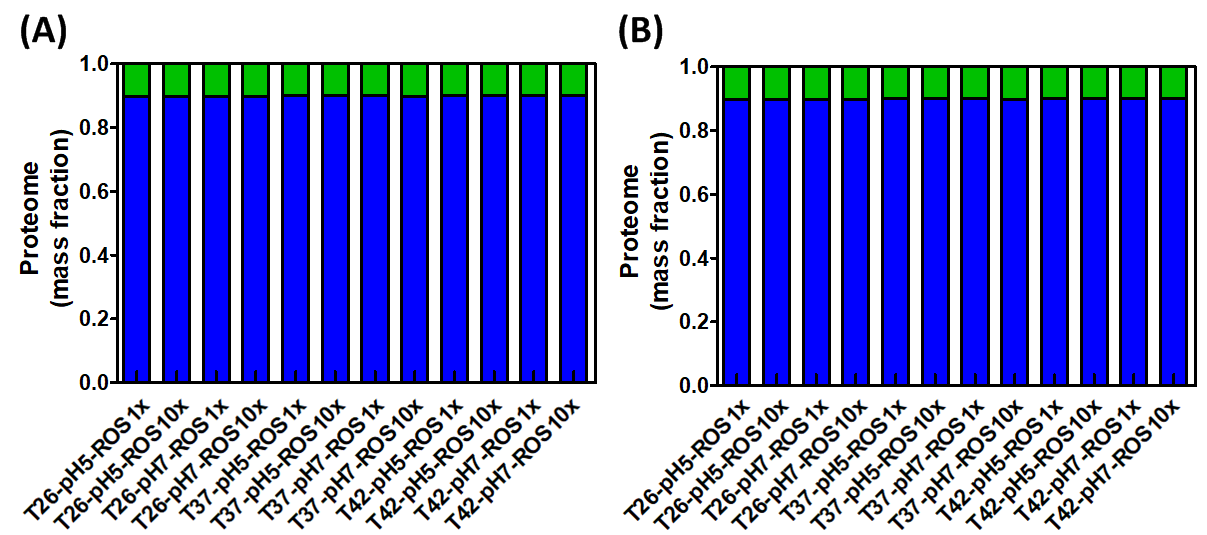
**

Fig B. Mass balance check for protein mass fractions. Under correct simulations, the total mass fraction of modeled (blue; 1578 proteins) and unmodeled proteins (green; 1 dummy protein in StressME) should sum to 1.0. Our simulations consistently reach the expected value of 1.0. (A) wild type strain. (B) Heat-evolved strain
