# Supplementary material for: StressME: Unified computing framework of Escherichia coli metabolism, gene expression, and stress responses
Source: PLoS Comput Biol. 2024 Feb 12;20(2):e1011865. doi: 10.1371/journal.pcbi.1011865 (PMC10890762; doi:10.1371/journal.pcbi.1011865)
Supplement: S6 Appendix — (DOCX) [file pcbi.1011865.s006.docx]

**S6 Appendix: Temperature-dependent phenotypes and proteome

*
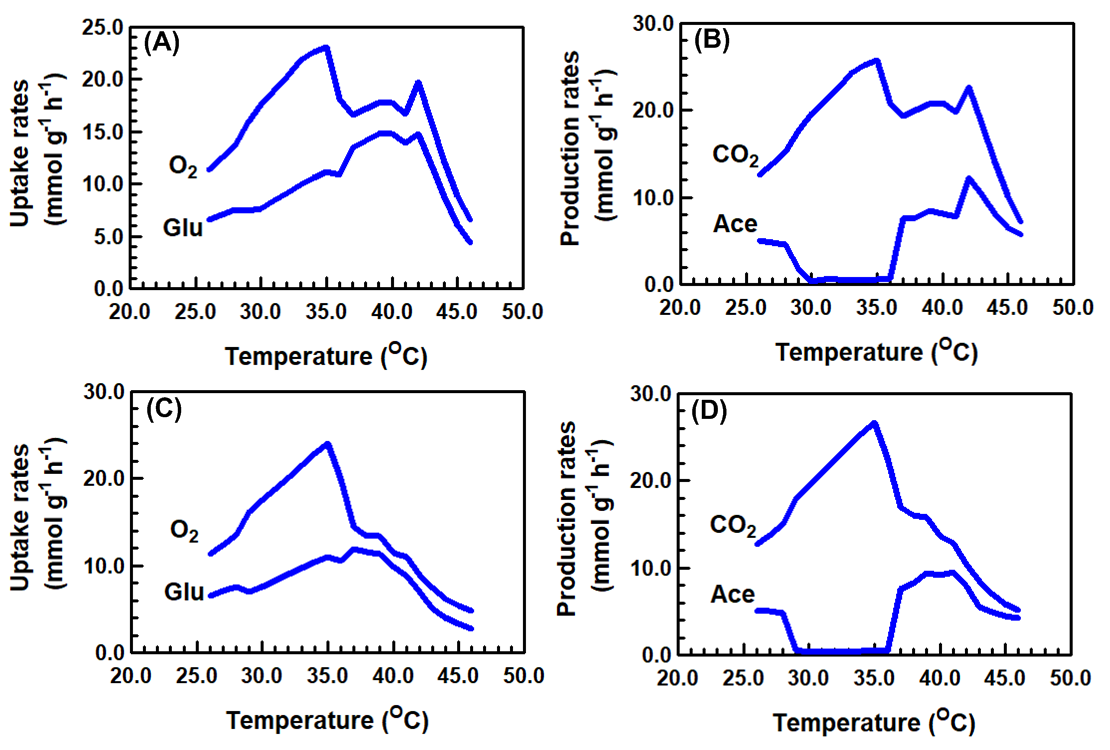
***

Fig A. StressME predicted exchange rates under thermal stress. (A) and (B): heat-evolved strain; (C) and (D): wild-type strain. Exchange rates shown are glucose (Glu) uptake rates, oxygen (O_2_) uptake rates, carbon dioxide (CO2) production rates (CO2) and acetate (Ace) production rates.

The analysis of the proteome reallocation for the heat-evolved strain at 32 and 40 ^o^C provides a global overview of how cells adjust their protein synthesis for different metabolic processes when exposed to environmental stress (Figure B). As compared to the proteome at 32 ^o^C, more proteome resources at 40 ^o^C were directed to synthesize protective chaperones (e.g., DnaJ, DnaK, GroS, GrpE) to assist folding and unfolding, sigma factor to regulate the stress response (e.g. rpoN and rpoH), and PPP (pentose phosphate pathway) proteins to maintain carbon homoeostasis (e.g. gnd, rpe and tktB). Meanwhile, cells at 40 ^o^C invested less proteome resources in energy production (i.e., TCA cycle with succinate dehydrogenase coupled to the electron transport chain) to reallocate the proteome resources into the protective proteins to tolerate the stress conditions.


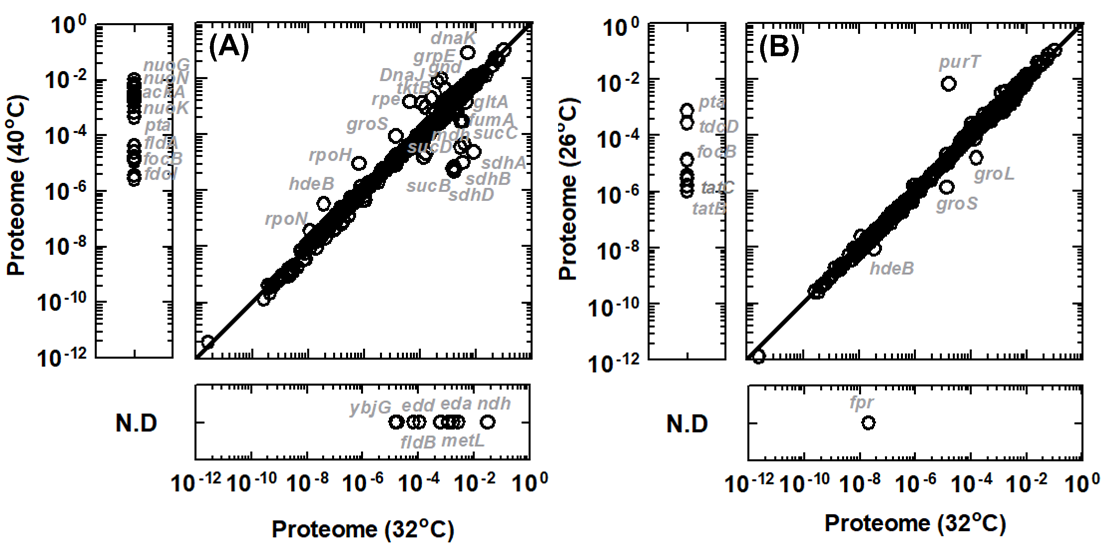


Fig B StressME predicted proteome reallocation at different temperatures (A) proteome at 32 ^o^C and 40 ^o^C (B) proteome at 32 ^o^C and 26 ^o^C
