# Supplementary material for: StressME: Unified computing framework of Escherichia coli metabolism, gene expression, and stress responses
Source: PLoS Comput Biol. 2024 Feb 12;20(2):e1011865. doi: 10.1371/journal.pcbi.1011865 (PMC10890762; doi:10.1371/journal.pcbi.1011865)
Supplement: S7 Appendix — (DOCX) [file pcbi.1011865.s007.docx]

**S7 Appendix: Alternative optima captured by StressME (purT vs. ackA)**

**Table A. Structure of three proteins with any one the three capable of catalyzing ACKr***


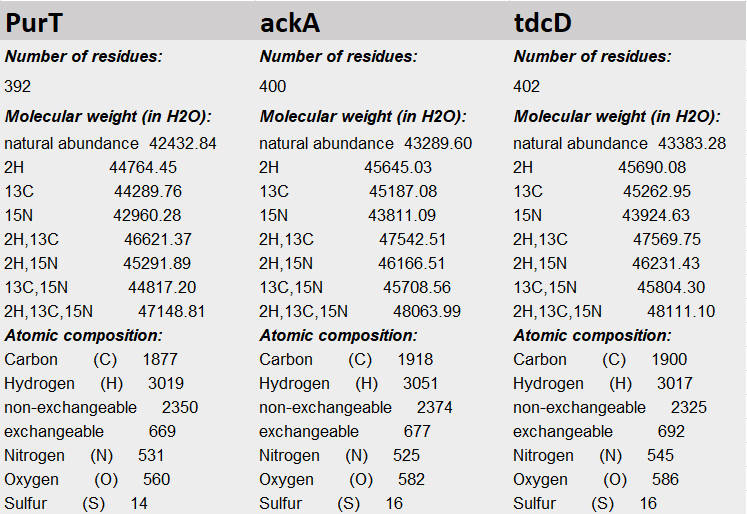


*Protein structure determined based on the amino acid sequence from https://spin.niddk.nih.gov/clore/


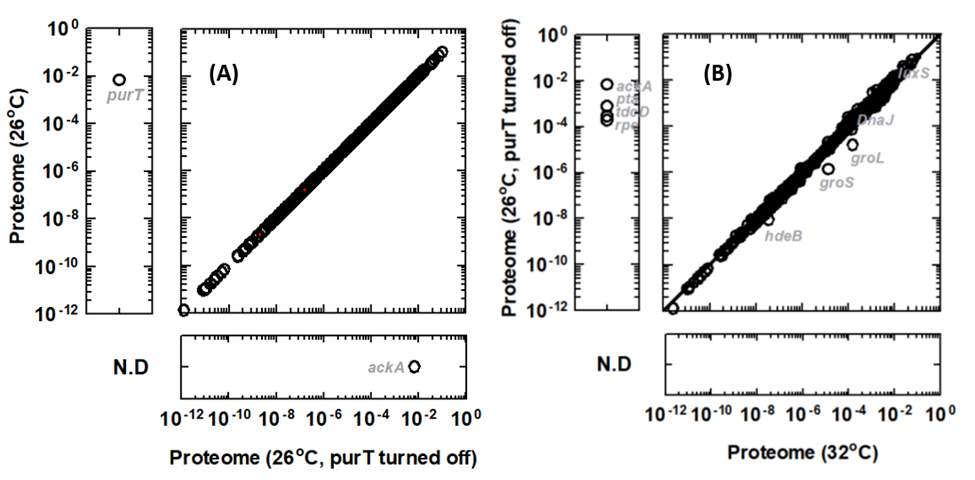


Fig A. Effect of purT and ackA translation and protein synthesis on overall proteome.
