# Supplementary material for: StressME: Unified computing framework of Escherichia coli metabolism, gene expression, and stress responses
Source: PLoS Comput Biol. 2024 Feb 12;20(2):e1011865. doi: 10.1371/journal.pcbi.1011865 (PMC10890762; doi:10.1371/journal.pcbi.1011865)
Supplement: S8 Appendix — (DOCX) [file pcbi.1011865.s008.docx]

**S8 Appendix: Reactions for NADH dehydrogenase and Quinolinate synthase**

**NADH dehydrogenase affected by ROS damage**

***(a) NADH16pp (NADH dehydrogenase coded by nuo)***

9.27286647516804e-7*mu NADH-DHI-CPLX_mod_2fe2s_mod_4fe4s_mod_fmn + 4.0 h_c + nadh_c + q8_c --> **-3.33823193106049*h2o2_c force_damage_NADH-DHI-CPLX_mod_2fe2s_mod_4fe4s_mod_fmn_h2o2** + **-3338.23193106049*o2s_c force_damage_NADH-DHI-CPLX_mod_2fe2s_mod_4fe4s_mod_fmn_o2s** + 3.0 h_p + nad_c + q8h2_c

***(b) NADH17pp (NADH dehydrogenase coded by nuo)***

9.27286647516804e-7*mu NADH-DHI-CPLX_mod_2fe2s_mod_4fe4s_mod_fmn + 4.0 h_c + mqn8_c + nadh_c --> **-3.33823193106049*h2o2_c force_damage_NADH-DHI-CPLX_mod_2fe2s_mod_4fe4s_mod_fmn_h2o2** + **-3338.23193106049*o2s_c force_damage_NADH-DHI-CPLX_mod_2fe2s_mod_4fe4s_mod_fmn_o2s** + 3.0 h_p + mql8_c + nad_c

**NADH dehydrogenase unaffected by ROS damage**

***(c) NADH5 (NADH dehydrogenase coded by ndh)***

5.65630208035663e-6*mu NADH-DHII-MONOMER_mod_mg2_mod_cu_mod_fad + h_c + nadh_c + q8_c --> nad_c + q8h2_c

**Quinolinate synthase affected by ROS damage**

***(d) QULNS (quinolinate synthase coded by nadA)***

0.01984448692471*mu CPLX0-7719_mod_4fe4s + dhap_c + iasp_c --> **-71440.1529289559*h2o2_c force_damage_CPLX0-7719_mod_4fe4s_h2o2** + **-71440152.9289559*o2s_c force_damage_CPLX0-7719_mod_4fe4s_o2s** + 2.0 h2o_c + pi_c + quln_c
