# Supplementary material for: StressME: Unified computing framework of Escherichia coli metabolism, gene expression, and stress responses
Source: PLoS Comput Biol. 2024 Feb 12;20(2):e1011865. doi: 10.1371/journal.pcbi.1011865 (PMC10890762; doi:10.1371/journal.pcbi.1011865)
Supplement: S9 Appendix — (DOCX) [file pcbi.1011865.s009.docx]

**S9 Appendix: StressME using Linux (clusters)**

**Installation**

# To set up virtual environment for python 3.6

virtualenv -p python MePython363
source MePython363/bin/activate

# install dependencies

**Accessories Version Installation**

1. cython 0.28.2 pip
2. sympy 1.1.1 pip
3. numpy 1.14.3 pip
4. scipy 1.1.0 pip
5. pytest 3.5.1 pip
6. pandas 0.22.0 pip
7. cycler 0.11.0 pip
8. matplotlib 2.2.2 pip
9. biopython 1.76 pip
10. qMINOS* 5.6 https://github.com/SBRG/solvemepy
11. cobrame StressME 1.1 https://github.com/QCSB/StressME

[l] ecolime StressME 1.1 https://github.com/QCSB/StressME

[m] oxidizeme StressME 1.1 https://github.com/QCSB/StressME

[n] acidifyme StressME 1.1 https://github.com/QCSB/StressME

[o] meuser StressME 1.1 https://github.com/QCSB/StressME

*See <https://github.com/SBRG/solvemepy> for qMINOS installation

**Simulations on Linux clusters by slurm**

*salloc --time=2:0:0 --ntasks=1 --cpus-per-task=1 --mem-per-cpu=8G --account=<your_account> python StressME_wildtype_keff.py 42 5.0 10*

or:

*sbatch --mem=8G --account=<your_account> --time=2:00:00 --output StressME_wildtype StressME_wildtype.sh*

where StressME_wildtype.sh is coded as:

#!/bin/bash

#SBATCH --time=2:00:00

#SBATCH --account=<your_account>

python StressME_wildtype_keff.py 42 5.0 10

Here “42 5.0 10” refers to the triple stress conditions at temperature 42 ℃, pH 5.0 and ROS 10X of the basal level.
