# Supplementary material for: StressME: Unified computing framework of Escherichia coli metabolism, gene expression, and stress responses
Source: PLoS Comput Biol. 2024 Feb 12;20(2):e1011865. doi: 10.1371/journal.pcbi.1011865 (PMC10890762; doi:10.1371/journal.pcbi.1011865)
Supplement: S10 Appendix — (DOCX) [file pcbi.1011865.s010.docx]

**S10 Appendix:** **StressME using docker**

Docker allows ME-model users to run StressME locally without going through the complicated processes of installing solvers and dependencies (SI-6) that may be incompatible with each other due to their version update.

The Docker image of StressME can be found on Docker Hub (queensysbio/stressme:v1.1). This build was developed from the modified version of COBRAme and EcoliME kernel to integrate FoldME and AcidifyME with OxidizeME. This build also includes qMINOS solver that users can use to solve StressME using solvemepy.

The installed COBRAme (StressME version), ECOLIme (StressME version), AcidifyME, OxidizeME and solvemepy packages can be found in /source/ after a new container has been created from the image of StressME. The working directory is /home/meuser, where ME-model users can run simulations and export output from the StressME container to the host. See scripts in <https://github.com/QCSB/StressME/meuser> for details.

**Installation on Windows Subsystem for Linux (WSL2)**

Steps to run a Docker container from the image of StressME with everything required to run the StressME using qMINOS:

1. Install Ubuntu on WSL2 on Windows 10/11.
   <https://ubuntu.com/tutorials/install-ubuntu-on-wsl2-on-windows-10#1-overview>
2. Download Docker Desktop for Windows <https://desktop.docker.com/win/main/amd64/Docker%20Desktop%20Installer.exe>
3. Start Docker Desktop from the Windows Start menu. From the Docker menu, select **Settings** and then **General**. Select the **Use WSL 2 based engine** check box. Select **Apply & Restart**
4. Pull the latest StressME image from DockerHub with:
   docker pull queensysbio/stressme:v1.1.

**Simulations on Windows Subsystem for Linux (WSL2)**

1. Start Docker Desktop from the Windows Start menu
2. Start another PowerShell window from the Windows Start menu. In the command line, type “ubuntu”
3. Type “cd /mnt/c/Users/<your user name in this local computer>/Desktop” and then type “mkdir StressME_wild_simulations” and “mkdir StressME_heat_simulations” to create host directories to receive output from the Docker StressME container
4. Type “cd StressME_wild_simulations” or “cd StressME_heat_simulations” and then type “docker run -p 8888:8888 --rm -i -v $(pwd):/mount_point/ -t queensysbio/stressme:v1.1 bash” . This will start the Docker StressME container (virtual machine) in the working directory: /home/meuser.
5. Type “ls” to check files available in the working directory.
6. Type “python StressME_wildtype_keff.py 42 5.0 10” or “python StressME_heatevolved_keff.py 42 5.0 10” to run simulations for the wild type or the heat evolved strains. Here “42 5.0 10” refers to the triple stress conditions at temperature 42 ℃, pH 5.0 and ROS 10X of the basal level.
7. After simulations are done, three csv files (TripleStressME_proteome.csv, TripleStressME_phenotypes.csv, and TripleStressME_fluxes.csv) are found in the working directory /home/meuser, representing the protein mass fractions, metabolic fluxes and phenotypes under the test stress conditions. Type “cp *.csv /mount_point/ StressME_wild_simulations” or “cp *.csv /mount_point/ StressME_heat_simulations” to export results to the corresponding host directories.
8. Use “CTRL+D” to exit Docker StressME container to go back to the host directory.
9. Type “explorer.exe .” to open File Explorer to view the csv data.

**Simulations on Jupiter notebook**

1. Start Docker Desktop from the Windows Start menu
2. In WSL2 or powershell, run ‘jupyter notebook --ip=0.0.0.0 --port=8888 --allow-root’
3. Copy the random token that is generated on the screen.
4. Paste the token on <http://localhost:8888/> to launch jupyter notebook
5. Run StressME_heatevolved_keff.ipynb and StressME_wildtype_keff.ipynb for simulations.
