# Supplementary material for: StressME: Unified computing framework of Escherichia coli metabolism, gene expression, and stress responses
Source: PLoS Comput Biol. 2024 Feb 12;20(2):e1011865. doi: 10.1371/journal.pcbi.1011865 (PMC10890762; doi:10.1371/journal.pcbi.1011865)
Supplement: Attachment — Submitted filename: Response to the editor and reviewers.docx [file pcbi.1011865.s011.docx]

**December 21, 2023**

**Editors**

**PLoS Computational Biology**

**Dear Dr. Medrano-Soto:**

We are submitting the revised manuscript entitled “StressME: unified computing framework of *Escherichia coli* metabolism, gene expression, and stress responses” by Jiao Zhao, Ke Chen, Bernhard O. Palsson and Laurence Yang to be considered for publication as an original research article in PLoS Computational Biology.

We sincerely thank the editors and reviewers for their valuable comments and suggestions, which have helped to significantly improve the quality of the revised version. We have considered all review comments carefully and tried our best to address them. Below are the original comments from reviewers in black and our responses in red.

We have prepared two versions of the revised manuscript: one with all changes highlighted in red and the other a clean version uploaded as the revised manuscript file.

We have also made the StressME github (https://github.com/QCSB/StressME) and dockerhub (https://hub.docker.com/r/queensysbio/stressme/tags) public.

We hope that the reviewers and the editors will consider our revised manuscript acceptable for publication in PLoS Computational Biology.

Thank you for considering our manuscript.

Sincerely,

Laurence Yang, Ph.D.

Assistant Professor

Department of Chemical Engineering

Queen's University

Kingston, ON K7L 3N6, Canada

**Response to Reviewer#1:**

We appreciate the reviewer for all valuable comments and suggestions, which have helped to significantly improve the quality of the manuscript. We have considered all review comments carefully. Below are the original comments from the reviewer in black and our responses in red.

**Major:**

(1) **In Introduction, the authors should enumerate specific scenarios where StressME could find applications. While it seems that information was provided in the last paragraph of the manuscript, it should be included in the Introduction to better justify the development of this tool.**

    We have expanded a paragraph in page 6 to indicate that StressME could find applications in industrial microbial processes (e.g. food industrial processes, waste treatment, and high-density cultivation confronted with multiple stressors) with references.
(2) **In the Quality control of the StressME model section, according to the authors, the parameters of the three individual ME models were obtained by fitting with experimental data, while the parameters of StressME were directly adapted from those models except the Keff of DXPRli. I wonder if the StressME parameters can be estimated in the same way.**

    We understand the logic behind the reviewer’s comment. We have tried to describe the Keff optimization more logically with more experimental data support.

    First, StressME, FoldME, AcidifyME, and OxidizeME are all E. coli ME models, describing metabolism and gene expression of E. coli under different stress conditions. Therefore, the parameters of the StressME can be directly adapted from individual E. coli ME models, whose parameters are based on experimental data. As such, the parameters of StressME are set using the same experimental data as the individual models.

    Second, figure 4 (page 39) shows the validation of the StressME with optimized Keffs against the experimental data.

    Third, based on a single effect sensitivity analysis (Figure 3 in page 38) for all metabolic reaction Keffs, we determined that the only parameter needing adjustment for StressME was DXPRli. The idea behind this optimization protocol is that we try to change a minimal number of Keffs that together should satisfy the different strain and stress conditions, considering the robustness of the complex networks.

    Finally, we found the biological evidence to support that the difference in DXPRli Keff may cause the heat-evolved strain to be distinct from the wild type strain (page 14). 1-deoxy-d-xylulose 5-phosphate reductoisomerase (DXPRLi coded by dxr) catalyzes the first committed step in the pathway of terpenoid biosynthesis, which is associated with a wide array of biomolecules for electron transport (ubiquinone and menaquinone) in respiration chain and membrane biosynthesis and stability. Therefore, more proteome resources in the wild type will be allocated to synthesize the key DXPRLi and its protective chaperones under thermal stress. Characterization of DXPRLi has shown that this enzyme can be heat stable up to 60 ℃ (Kuzuyama et al., 2000). Thus, the DXPRli in the heat-evolved strain may not need more proteome resources to synthesize itself and its chaperone under higher temperature, thereby saving more resources for other protein synthesis that can support growth.

    **Reference**

    *Kuzuyama T, Takahashi S, Takagi M, Seto H. Characterization of 1-deoxy-D-xylulose 5-phosphate reductoisomerase, an enzyme involved in isopentenyl diphosphate biosynthesis, and identification of its catalytic amino acid residues. J Biol Chem. 2000;275(26):19928-32*
(3) **In the Keff in single- and Integrated-StressME models section, why the Keffs in FoldME is different from AcidifyME/OxidizeME? The authors states that “This causes more protective chaperones to be produced resulting in additional proteome stress that decreases growth rates at high temperature (Figure 2C) or at low pH (Figure 2D).”, however, model with FoldME keffs requires more protein resources based on Fig. 2E and 2F.**

    The reviewer is correct in observing that the model with FoldME keffs can express more proteins that are not chaperones. The confusion here is that the higher protein expression (of non-chaperones) does not indicate the requirement for more protein resources, given the constraint on total proteome mass. Instead, it shows that the FoldME keff model has the capacity to express more other useful proteins (besides chaperones). As such, it can be more efficient at growing under heat or acid stress (Figure 2C and 2D). In other words, there is more room to reallocate the proteome toward proteins that increase growth rate.

    A key reason for the increased protein allocation capacity is that the Dxr protein can be expressed at a much lower (around 8872-fold decrease) concentration (Eq.1) using the FoldME Keffs (88.72 s^-1^) vs. the wild type Keffs (0.01 s^-1^). Therefore, other proteins can be expressed at higher concentrations when Dxr and its protective chaperones (dnaK and dnaJ) for folding are expressed at lower concentrations in the FoldME Keff model, given the constraint on total proteome mass.

    We have re-written the paragraph at page 11 to avoid the confusion.
(4) **In the Phenotype validation using consolidated kinetome section, the authors states that “it confirms the overall robustness of StressME”. Does it suggest that the StressME is insensitive to gene/reaction addition/deletion and parameter change?**

    This is correct – using sensitivity analysis, we showed that if every Keff is changed, one at a time, the model predictions (including phenotype, proteome, etc.) are insensitive. The exception was DXPRli, which we investigated in detail.

Therefore, we suggest that, generally, even if a Keff parameter is changed for a single gene/reaction, it is likely that the overall model predictions are insensitive due to the robustness of complex networks.

However, we do not mean to say that if a gene/reaction were added/deleted, then the model predictions would not change. E.g., if a single essential gene is deleted, we know the model predictions change drastically (i.e., zero growth rate).

We mean that we do not need to re-estimate all other Keffs again if a new gene/reaction is added.

We have revised the text to clarify this point.

**Original:**

“…only minimum work is required for parameter coordination if more genes, proteins, metabolites, and reactions are added to the model architecture.”

**Revised (page 16):**

“…if more genes, proteins, metabolites, and reactions are added to the model, re-fitting the Keffs for all other reactions is likely not required.”

(5) **In Methods, reference should be provided to clarify “three single stress ME models (FoldME, OxidizeME and AcidifyME) were rebuilt from the same starting point (E. coli K-12 MG1655 ME-model iJL1678-ME) under Python version 3.6.3 based on the published protocols”.**

We have added references to Methods (page 27) for three single stress ME models (FoldME[Chen et al., 2017], OxidizeME[Yang et al., 2019] and AcidifyME[Du et al., 2019]), which were rebuilt from the same starting point (E. coli K-12 MG1655 ME-model iJL1678-ME[LlLloyd et al., 2018]) under Python version 3.6.3 based on the published protocols[ LlLloyd et al., 2018]

**Reference**

*LlLloyd CJ, Ebrahim A, Yang L, King ZA, Catoiu E, O'Brien EJ, et al. COBRAme: A computational framework for genome-scale models of metabolism and gene expression. Plos Computational Biology. 2018;14(7).*

*Chen K, Gao Y, Mih N, O'Brien EJ, Yang L, Palsson BO. Thermosensitivity of growth is determined by chaperone-mediated proteome reallocation. Proceedings of the National Academy of Sciences of the United States of America. 2017;114(43):11548-53.*

*Du B, Yang L, Lloyd CJ, Fang X, Palsson BO. Genome-scale model of metabolism and gene expression provides a multi-scale description of acid stress responses in Escherichia coli. Plos Computational Biology. 2019;15(12).*

*Yang L, Mih N, Anand A, Park JH, Tan J, Yurkovich JT, et al. Cellular responses to reactive oxygen species are predicted from molecular mechanisms. Proceedings of the National Academy of Sciences of the United States of America. 2019;116(28):14368-73.*

(6) **Is there a maximum limit for total protein allocation used in the model development? If so, how the authors determined this value and is it kept constant or variable in the different stress conditions tested?**

    The individual protein amount in the ME model is expressed as P_j_ in the unit of g protein /gDCW cell, and the total modeled protein amount is expressed as P_t_.

Considering that the protein synthesized is diluted by cell division, we have:

$P_{j}= \frac{\left( {MW}_{j}\times V_{trsl, j} \right)}{\mu}$	(Eq. 1)

$P_{t}= \sum_{j} \frac{\left( {MW}_{j}\times V_{trsl, j} \right)}{\mu}$	(Eq. 2)

We have individual protein mass fraction P_j,mf_:

$P_{j,mf}= \frac{P_{j}}{P_{t}}$	(Eq. 3)

Of that total protein mass, we allow f_modeled (=1 – f_unmodeled) mass fraction to be allocated towards the modeled proteins, where f_unmodeled represents the “dummy protein” in the model that is set using the “unmodeled_protein_fraction” model parameter (set to 10% in this work). The f_modeled value (90%) is close to what has been experimentally quantified for the proteome mass in E. coli (>95%) (Schmidt et al., 2016), and it has been increased from the previous ME models (e.g. 85% in OxidizeME) (Yang et al., 2019) considering more proteins were added to the StressME. Under any conditions, f_modeled + f_unmodeled should always be equal to 1 as a constant constraint for the total proteome.

**Reference**

*Schmidt A, Kochanowski K, Vedelaar S, Ahrne E, Volkmer B, Callipo L, et al. The quantitative and condition-dependent Escherichia coli proteome. Nat Biotechnol. 2016;34(1):104-10.

Yang L, Mih N, Anand A, Park JH, Tan J, Yurkovich JT, et al. Cellular responses to reactive oxygen species are predicted from molecular mechanisms. Proceedings of the National Academy of Sciences of the United States of America. 2019;116(28):14368-73.*

(7) **What is the computation time of running the StressME model?**

    Average time was estimated for the StressME running in local desktop (Intel(R) Core (TM) i7-10875H @ 2.30 GHz, 8 cores, 32GB DDR4-2933MHz) and Compute Canada Graham clusters (2 x Intel E5-2683 v4 Broadwell @ 2.1GHz, 1 CPU and 8 GB memory per task) under different stress conditions. The typical computation time is now listed in S4 Appendix in page 36 for the relevant use cases of the StressME model.
(8) **The software is not available according to the github URL provided.**

    We have made data publicly available by:

    (a) github URL has been set from private to public.
    <https://github.com/QCSB/StressME>
    (b) The Docker image of StressME can be downloaded on Docker Hub (https://hub.docker.com/r/queensysbio/stressme/tags).

**Minor:**

(1) **The structure of three proteins (tdcD, purT and ackA) should be in SI-4 rather than Table S2.**

    Thank you for catching that. We have revised the text to show that this information is listed in Table S8 in S7 Appendix.
(2) **In the heat adaptation cannot induce cross-protection against acid-involved stress section, reference should be provided for the statement “This shift requires pyruvate formate lyase (tdcE), which is inactive at pH7.”**

Thank you for highlighting a point that may cause confusion to future readers. This statement is based on StressME simulations. We have revised the text to clarify this point with some additional reference support (page 25).

In summary, the StressME simulations suggest that the heat-evolved strain may change the metabolism under acid stress from fully respiratory through TCA to respiro-fermentative metabolism through anaerobic glycolysis by way of pyruvate lyase (tdcE). We have found publications to support that the respiro-fermentative metabolism can be activated in *E. coli* under both aerobic and anaerobic conditions [Simensen et al., 2022; Tack et al., 2018; Knappe et al., 1999; Conradt et al., 1984]. The possible reason for the shift identified in the heat-evolved E. coli by StressME is that the heat-evolved strain may use the proteome-ready-to-deploy respiro-fermentative pathways [Dinh et al., 2023] to avoid the proteome limitation for energy biogenesis under acid stress, because tdcE is constitutively expressed in *E. coli* [Crain et al., 2014] and because the proteome cost for energy biogenesis is less expensive by fermentation than by respiration in *E.coli* [Basan et al., 2015]*.* These two reasons justify the choice of the respiro-fermentative metabolism under proteome constraints in the face of acid stress. Further experimental work is required to validate the simulated switch between TCA and respiro-fermentative metabolism under different stress conditions.

**Reference**

*Simensen V, Schulz C, Karlsen E, Bratelund S, Burgos I, Thorfinnsdottir LB, et al. Experimental determination of Escherichia coli biomass composition for constraint-based metabolic modeling. PLoS One. 2022;17(1): e0262450.*

*Tack I, Nimmegeers P, Akkermans S, Logist F, Van Impe JFM. A low-complexity metabolic network model for the respiratory and fermentative metabolism of Escherichia coli. PLoS One. 2018;13(8):e0202565.*

*Knappe J, Sawers G. A radical-chemical route to acetyl-CoA: the anaerobically induced pyruvate formate-lyase system of Escherichia coli. FEMS Microbiol Rev. 1990;6(4):383-98.*

*Conradt H, Hohmann-Berger M, Hohmann HP, Blaschkowski HP, Knappe J. Pyruvate formate-lyase (inactive form) and pyruvate formate-lyase activating enzyme of Escherichia coli: isolation and structural properties. Arch Biochem Biophys. 1984;228(1):133-42

Dinh HV, Maranas CD. Evaluating proteome allocation of Saccharomyces cerevisiae phenotypes with resource balance analysis. Metab Eng. 2023;77:242-55.*

*Crain AV, Broderick JB. Pyruvate formate-lyase and its activation by pyruvate formate-lyase activating enzyme. J Biol Chem. 2014;289(9):5723-9.*

*Basan M, Hui S, Okano H, Zhang Z, Shen Y, Williamson JR, et al. Overflow metabolism in Escherichia coli results from efficient proteome allocation. Nature. 2015;528(7580):99-104*

**Response to Reviewer#2:**

We appreciate the reviewer for all valuable comments and suggestions, which have helped to significantly improve the quality of the manuscript. We have considered all review comments carefully. Below are the original comments from the reviewer in black and our responses in red.

**Major:**

(1) **Throughout the paper, it is not clear at all what are the differences between the model from STRESSME and the previous one coming from FoldME, AcidifyME, and OxidizeME. Despite the authors indicating the number of genes and reactions for every reconstruction, still lacks how many reactions are included in the last database that shares and excludes reactions. A global landscape of the differences and similarities can be useful for the reader. Based on the text, seems like the model iZY1689-StressME has close similarity in reconstruction concerning the previous version. Why did iZY1689-StressME have a lower number of proteins (1,568 proteins) than the previous one (for instance 1,578 proteins), some of them were deleted?**

    Thank you for highlighting a point that may cause confusion to future readers. A table listing reactions added to the StressME after every reconstruction step shown in Figure 1 has now been provided in Table S2 (S1 Appendix page 35). Detailed information about the added reactions with chemical equations can be found in S2 Appendix (page 35).

iZY1689-StressME has a higher number of proteins (1,578 proteins) than the previous one (for instance 1,578 proteins) (Figure 1 and Table S1 in S1 Appendix). From the new added Table S2 (S1 Appendix) and reactions with chemical equations (S2 Appendix), 24,080 more reactions have been added to StressME, significantly expanding the reconstruction as compared with the previous versions.

(2) **It should be convenient to evaluate the quality of the reconstruction with tools like MEMOTE (**[**https://www.nature.com/articles/s41587-020-0446-y**](https://www.nature.com/articles/s41587-020-0446-y)**). This can be useful to identify the quality of the global reconstruction.**

    That is an interesting point to assess the quality of StressME by MEMOTE. However, we have found that MEMOTE currently accepts genome-scale metabolic models (M-models) in SBML3FBC format as input, which the CobraME package to build StressME does not support. In the next version of StressME, we will try to support the SBML3FBC standard so that MEMOTE can also assess the quality of Metabolic and Expression Models (ME-models).

For this revision, we further assessed the quality of the StressME model across all test conditions by checking the mass balance for metabolome and proteome (S5 Appendix in page 36). The assessment indicates a perfect match between the consumption and production of each metabolite, as well as between the simulated and total protein mass fraction, confirming the robustness of complex networks and algorithms used for StressME.

(3) **In terms of the metabolic responses, is it possible the existence different strategies to face the set of stresses? How robust are these mechanisms?**

    That is a good point. We have discussed the potential different strategies to face different stressors in the original “Quality control of the StressME model” section. Since it is an interesting topic, we have moved these contents to a subtitle “Alternative optima (strategies) to face stressors” starting from page 16.

    (a) Page 16: For those observable metabolic fluxes to support 95%-100% of maximal biomass production rates, the flux variability analysis (FVA) found the boundedness of the optimal solution space, with narrow ranges by fixing 100% growth rates. It suggests limited alternative optima that can be obtained by StressME, confirming the robustness of the model.

    (b) Page 17: Alternative optima was captured by StressME (i.e., TdcD, PurT and AckA with any one capable of catalyzing the reaction ACKr). However, the protein structure, stability, and inactivation analysis suggest that the purT expression preferable to ackA is due to the alternative optima captured by StressME, rather than the ‘hidden’ biological mechanism that may significantly affect the overall proteome and fluxome.

Moreover, in the “Investigating cross-stress and cross-talk resistance using StressME” section (page 23 and SI-7):

(c) StressME simulations indicate that cells tried to use ndh-coded NADH dehydrogenase to avoid the uneconomical nuo-coded NADH pathway vulnerable to the ROS damage. This potential mechanism can be considered as a reasonable alternative strategy to respond to stressors.

(4) **How do you deal with the feedback mechanisms between stress and metabolism? To my understanding, this is not included in the model. If included the model can improve their predictions?**

We agree that the inclusion of the feedback mechanisms can further improve the model predictions. In the paper, we have touched some preliminary feedback mechanisms, such as:

(a) a feedback mechanism between oxidative stress and NADH dehydrogenase: as ROS level increased, cells tried to use ndh-coded NADH dehydrogenase to avoid the uneconomical nuo-coded NADH pathway vulnerable to the ROS damage (page 23 and S8 Appendix)
(b) a feedback between acid stress and metabolism switch (page 24 and Figure 9 at page 44): as pH decreased, the heat-evolved strain changed the metabolism from fully respiratory through TCA to respiro-fermentative metabolism through anaerobic glycolysis. The respiro-fermentative metabolism has been reported to be “ready-to-deploy” pathways by proteome reallocation (Dinh et al., 2023), and more economical for energy biogenesis than respiration under stress conditions (Basan et al., 2015).

We have mentioned such feedback mechanisms between stress and metabolism in the revision. Further improvement on the StressME with respect to feedback mechanism may include the mechanism of how stress signals can be received by sensor proteins and how metabolomes of stressed *E. coli* can cooperate with proteome and fluxome to collectively response to different stressors upon signal reception.

**Reference**

*Basan M, Hui S, Okano H, Zhang Z, Shen Y, Williamson JR, et al. Overflow metabolism in Escherichia coli results from efficient proteome allocation. Nature. 2015;528(7580):99-104*

*Dinh HV, Maranas CD. Evaluating proteome allocation of Saccharomyces cerevisiae phenotypes with resource balance analysis. Metab Eng. 2023;77:242-55.*

(5) **In terms of equation 1 and the optimization of the Keff vectors. How many parameters are included in the model? Keff represent a vector, one for each reaction in the reconstruction, or only was subjected to a few of them? Will be useful to explain that point. In addition, equation 1 seems a little counterintuitive when including the keff. Eq. 1 indicates that if a Keff value is increased, the amount of the protein to be synthesized is decreased. is it explained by a low effective production of the enzyme that functionally does not perfectly complement its enzymatic work? This assumption is valid for all the enzymes in the reconstruction? please explain.**

    The Keffs optimization was implemented by computing growth rates when each metabolic reaction Keff of total 5266 metabolic reactions (that is, a vector of 5266 Keffs) was altered at one time from default (wild-type) to alternative (heat-evolved) Keff. All simulated 5266 growth rates were then sorted, and the most influential reactions subject to Keff change were identified. Based on this single effect sensitivity analysis (Figure 3 in page 38) for all metabolic reaction Keffs, we determined that the only parameter needing adjustment for StressME was DXPRli. The idea behind this optimization protocol is that we try to change a minimal number of Keffs that together should satisfy the different *E.coli* strain and stress conditions, considering the robustness of the complex networks.

    In the revision, we have also added the biological evidence to support that the difference in DXPRli Keff may cause the heat-evolved strain to be distinct from the wild type strain (page 14). 1-deoxy-d-xylulose 5-phosphate reductoisomerase (DXPRLi coded by dxr) catalyzes the first committed step in the pathway of terpenoid biosynthesis, which is associated with a wide array of biomolecules for electron transport (ubiquinone and menaquinone) in respiration chain and membrane biosynthesis and stability. Therefore, more proteome resources in the wild type will be allocated to synthesize the key DXPRLi and its protective chaperones under thermal stress. Characterization of DXPRLi has shown that this enzyme can be heat-stable up to 60 ℃ (Kuzuyama et al., 2000). Thus, the DXPRli in the heat-evolved strain may not need more proteome resources to synthesize itself and its chaperone under higher temperature after the strain adaptation, thereby saving more resources for other protein synthesis that can support growth under thermal stress.

Keff is defined as effective turnover rate of enzyme(s) (i.e., number of substrate molecules converted to product molecules per enzyme molecule in a unit time). Therefore, less amount of enzyme is required to be synthesized if Keff value is high (Eq. 1), because the corresponding enzyme is more efficient in catalysis of the reaction.

$V_{translation}=\left( \frac{Keff}{\mu} \right)V_{metabolic\_reaction}$	(Eq. 1)

Eq. 1 is valid for all enzymes in the reconstruction (Lloyd et al., 2018).

**Reference**

*Kuzuyama T, Takahashi S, Takagi M, Seto H. Characterization of 1-deoxy-D-xylulose 5-phosphate reductoisomerase, an enzyme involved in isopentenyl diphosphate biosynthesis, and identification of its catalytic amino acid residues. J Biol Chem. 2000;275(26):19928-32

Lloyd CJ, Ebrahim A, Yang L, King ZA, Catoiu E, O'Brien EJ, et al. COBRAme: A computational framework for genome-scale models of metabolism and gene expression. Plos Computational Biology. 2018;14(7).*

(6) **How does keff from the previous model change to the one obtained from STRESSME? The profiles of new keff are unique to obtain the optimization?**

    Two single Keff vectors were optimized for the StressME representing the wild-type and heat-evolved strains, respectively. This optimization is based on a protocol to change a minimal number of Keffs that together satisfy the different strain and stress conditions, considering the robustness of complex networks. This was implemented by computing growth rates when each metabolic reaction Keff of total 5266 metabolic reactions was altered at one time from default (wild-type) to alternative (heat-evolved) Keff. All simulated 5266 growth rates were then sorted, and the most influential reactions subject to Keff change were identified. Based on this single effect sensitivity analysis (Figure 3 in page 38) for all metabolic reaction Keffs, we determined that the only parameter needing adjustment for StressME was DXPRli. Please see our response to comment #4 regarding the biological meaning of the change of the DXPRli Keff in the heat-evolved strain.

    Except for the most influential reaction subject to Keff change (e.g., DXPRli), Keffs for other reactions can be non-unique values (e.g., Keffs can change from the wild type to the heat-evolved) without significant effect on the growth rates (Figure 3A in page 38), suggesting that these Keff values may already be in a reasonable range, or some reactions may have alternate pathways to fulfill similar functions.
(7) **When the authors say: " we identified incompatibilities between the Keff values used in FoldME and AcidifyME: swapping their Keff values produced simulations inconsistent with published results for thermal and acid responses (Figure 2C-D). " How is that possible if the networks seem to be very similar as the authors indicate in the phrase "The resulting model, called iZY1689-StressME, comprises 1,689 genes, 1,578 proteins, 1,673 metabolites, 1,692 complexes and 36,735 reactions (Fig. 1). This biological scope is a significant expansion over the original iJL1678b-ME composed of 1,678 genes, 1,568 proteins, 1,671 metabolites, 1,526 complexes and 12,655 reactions." is this considered a significative expansion of the previous model? Pls explain.**

    Thank you for highlighting a point that may cause confusion to future readers. The StressME has 1578 proteins and 36,735 reactions, a significant expansion over the original iJL1678b-ME composed of 1568 proteins and 12,655 reactions. The added 24,080 reactions are relevant to protein folding and unfolding, demetallation of Fe (II) proteins by ROS, mismetallation by alternative metal ions, oxidization, and repair of Iron–sulfur clusters, unincorporated Fe (II) reaction with H_2_O_2_ (Featon reaction) to generate hydroxyl radicals, and the protection from Dps protein to store unincorporated Fe (II). Therefore, the change of key Keffs for most influential reactions would significantly change the expression of the proteins for metabolic functions, and then change the expression of the protective proteins (e.g., chaperones) to stabilize these proteins under stress conditions. In other words, the difference in Keff may cause a reallocation of the proteome over the intracellular network, given the total protein abundance constraint, thereby affecting the cellular metabolism.

    It should also be noted that FoldME is a ME model for the heat-evolved strain, whereas OxidizeME or AcidifyME is the one for the wild type strain. It is reasonable that there are incompatibilities between the Keff values used in individual ME models. In this work, we tried to coordinate the Keff values so that a change of a minimal number of Keffs should together satisfy the different strain and stress conditions, considering the robustness of the complex networks. Based on single effect sensitivity analysis (Figure 3 in page 38) for all metabolic reaction Keffs, we determined that the only parameter needing adjustment for StressME was DXPRli. Keffs for other reactions can be non-unique values (e.g., Keffs can change from the wild type to the heat-evolved) without significant effect on the growth rates (Figure 3A in page 38), suggesting that these Keff values may already be in a reasonable range, or some reactions may have alternate pathways to fulfill similar functions.
(8) **Figure 5, how can be explained the increase of variability as the temperature increases and the systems look for sub-optimal conditions in growth rate? Is there an explanation for this behavior?**

    As temperature increases, there is a boost of the protective proteins (e.g., chaperones) to stabilize the important proteins for metabolic functions. It will cause the reallocation of the proteome over the intracellular metabolic network, causing more flexible metabolic response to the thermal stress to meet the constraint of the total proteome available for allocation. Moreover, alternative strategies may be available to respond to the high temperature stress (page 24 and Figure 9 at page 44), increasing the variability in metabolism and sub-optimal growth status.
(9) **As the authors argued, StressME is capable of exploring the crosstalk among these external perturbations (thermal, oxidative, and acid stress), however at the end of the paper, the authors explore only two conditions associated with situations where both stimuli are applied by consecutive order. Thinking more about the crosstalk mechanism, what occurs if two or three stresses are applied simultaneously? What are the predictions of the model? Sketch this situation can be useful for the reader.**

    Thank you for highlighting a potential area that needs to be focused on. As matter of fact, we have considered two scenarios by consecutive order to reconstruct the wild-type and heat-evolved strains, respectively, and then we have applied dual or triple stress simultaneously but we have not explicitly stated these crosstalk mechanisms.

    We have touched the crosstalk mechanisms in:

    Figure 7 (thermal and oxidative dual stress, page 24 and 42);
    Figure 8 and Figure 9 (thermal and acid dual stress, page 26, 43 and 44);
    Figure 10 (oxidative and acid dual stress, page 26 and 45);
    Figure 11 (thermal-oxidative-acid triple stress, page 27 and 46)

Since the StressME studies on crosstalk protection were not clearly stated in both the subtitle and the text body, we have modified the subtitle (page 20) and added an introducing (page 20) and summary paragraph (page 27) before and after these studies.
